# Intradural fat graft packing is not indispensable in preventing postoperative cerebrospinal fluid leakage in endoscopic endonasal pituitary adenoma surgeries

**DOI:** 10.3389/fonc.2023.1222581

**Published:** 2023-07-26

**Authors:** Xiefeng Wang, Binbin Wang, Gang Cheng, Yongping You, Chao Tao

**Affiliations:** Department of Neurosurgery, The First Affiliated Hospital of Nanjing Medical University, Nanjing, China

**Keywords:** pituitary tumors, surgery, neuroendoscopy, cerebrospinal fluid leakage, skull base reconstruction

## Abstract

**Objectives:**

Is intradural fat graft packing indispensable in preventing postoperative cerebrospinal fluid leakage in endoscopic endonasal pituitary adenoma surgeries? This study aimed to review the methods and outcomes of our graded sellar floor reconstruction strategy without fat graft packing in endoscopic endonasal pituitary adenoma surgeries.

**Methods:**

From March 2018 to December 2022, 200 patients underwent endoscopic endonasal pituitary adenoma resection by a single author in our institute. We applied different graded skull base reconstruction strategies in different periods. Intradural fat graft packing was used to reconstruct the skull base in the early period, from March 2018 to June 2019, but fat graft was not used in the late period, from January 2020 to December 2022. The effect of these different graded skull base reconstruction strategies and whether intradural fat graft packing is necessary were evaluated by observing the incidence of postoperative cerebrospinal fluid leak.

**Results:**

In the early period, fat graft was used to reconstruct skull base when the intraoperative cerebrospinal fluid (CSF) leakage existed. There were two patients who suffered from postoperative cerebrospinal fluid leak in this group. In the late period, fat graft was not used to reconstruct the skull base, and no patient suffered from postoperative cerebrospinal fluid leakage in this group.

**Conclusions:**

Intradural fat graft packing is unnecessary in the endoscopic endonasal pituitary adenoma resection. The outcome of our graded sellar floor reconstruction strategy is satisfactory.

## 1 Introduction

Endoscopic endonasal approach (EEA) is becoming the main surgical approach for pituitary adenoma resection (1–5). EEA can provide better visualization and wider exposure than the traditional microscopic trans-sphenoidal approach (MTA) pituitary adenoma resection (6–8).

Skull base reconstruction is a critical step in endoscopic transnasal pituitary adenoma surgeries. The method of skull base repair varies among surgeons (2, 4, 5, 9–11). Many surgeons perform autologous fat graft packing during skull base reconstruction, which is effective but may also bring problems. There is no conclusion on whether fat packing is inevitable for skull base repair after pituitary adenoma surgery. We have applied different strategies of skull base reconstruction at different periods. Through retrospective analysis, we compare the efficacy of the two strategies to explore whether intradural fat graft packing is inevitable during skull base reconstruction.

From March 2018 to December 2022, a total of 200 patients underwent EEA pituitary adenoma resection by a single author in our institute. Different skull base reconstruction strategies were applied in different periods. In the early period, from March 2018 to June 2019, fat graft was used to reconstruct the skull base. In the late period, from January 2020 to December 2022, fat graft was not used. This study respectively reviewed the surgical methods and outcomes of these cases to find out if intradural fat graft packing is indispensable in preventing postoperative cerebrospinal fluid leakage in endoscopic endonasal pituitary adenoma surgeries.

## 2 Methods

### 2.1 Patients

In the early period, from March 2018 to June 2019, 75 patients underwent EEA pituitary adenoma resection in our institution. Of the 75 patients, 43 (57.3%) were male, 32 (42.7%) were female. According to the size of the tumor, 6 (8.0%) patients had a microadenoma (<1cm), 52 (69.3%) patients had a macroadenoma (≥1cm, ≤2.5cm), and 17 patients (22.7%) had a giant adenoma (>2.5cm). There were 19 (25.3%) patients who suffered from pituitary adenoma with cavernous sinus (CS) and (or) suprasellar invasion. A total of four patients suffered from recurrent pituitary adenoma in this group, and these patients had undergone transsphenoidal pituitary adenoma surgery before.

In the late period, from January 2020 to December 2022, a total of 125 patients underwent EEA pituitary adenoma resection in our institution. Of the 125 patients, 68 (54.4%) were male and 57 (45.6%) were female. According to the size of the tumor, 12 (9.6%) patients had a microadenoma (<1cm), 68 (54.4%) patients had a macroadenoma (≥1cm, ≤2.5cm), and 45 patients (36.0%) had a giant adenoma (>2.5cm). There were 29 (23.2%) patients who suffered from pituitary adenoma with cavernous sinus (CS) and (or) suprasellar invasion. A total of eight patients suffered from recurrent pituitary adenoma in this group, and these patients had undergone transsphenoidal pituitary adenoma surgery before.

### 2.2 Surgical technique

All patients underwent EEA to remove pituitary adenomas. The preoperative evaluation, intraoperative positioning, and tumor resection methods are consistent with the descriptions in our previous article (12). All the patients underwent postoperative pituitary function assessments on the first postoperative day. An MRI scan with contrast was performed within 3 days or after 3 months.

#### 2.2.1 Skull base reconstruction strategies in the early period

If intraoperative CSF leak did not exist, only artificial dura matter has been placed intradural. If the intraoperative CSF leak existed without visible arachnoid defect, inlayer fat graft and artificial dura matter were applied. If the intraoperative CSF leak existed with visible arachnoid defect, inlayer fat graft, and artificial dura matter, combined onlayer fascia lata graft were used. If intraoperative suprasellar space, third ventricle, or internal carotid artery (ICA) was exposed, inlayer fat graft and artificial dura matter, onlayer fascia lata graft, and pedicled nasoseptal flap were used.

The nasal cavity was usually packed for 3 days. If the fascia lata graft and pedicled nasoseptal flap were used, the nasal cavity was packed for a week.

#### 2.2.2 Skull base reconstruction strategies in the late period

Artificial dura mater was placed intradural in all the patients. However, intradural fat graft or muscle patty packing and dura suturing were not performed. Artificial bone or autologous bone fragments were not used.

The reconstruction methods for the epidural layer of the skull base are as follows.

First, free nasal septum mucosa flap or middle turbinate mucosa flap was used in the following situations: when there is no apparent cerebrospinal fluid leakage during the surgery; when a small amount of intraoperative cerebrospinal fluid leakage was observed, but there were no obvious tears on the arachnoid membrane and sellar diaphragm; and when an evident intraoperative cerebrospinal fluid leak was observed, with apparent tears on the arachnoid membrane and sellar diaphragm, meantime, the free nasal mucosa flap can completely cover the dura defect of the sellar floor.

**Figure 1:**
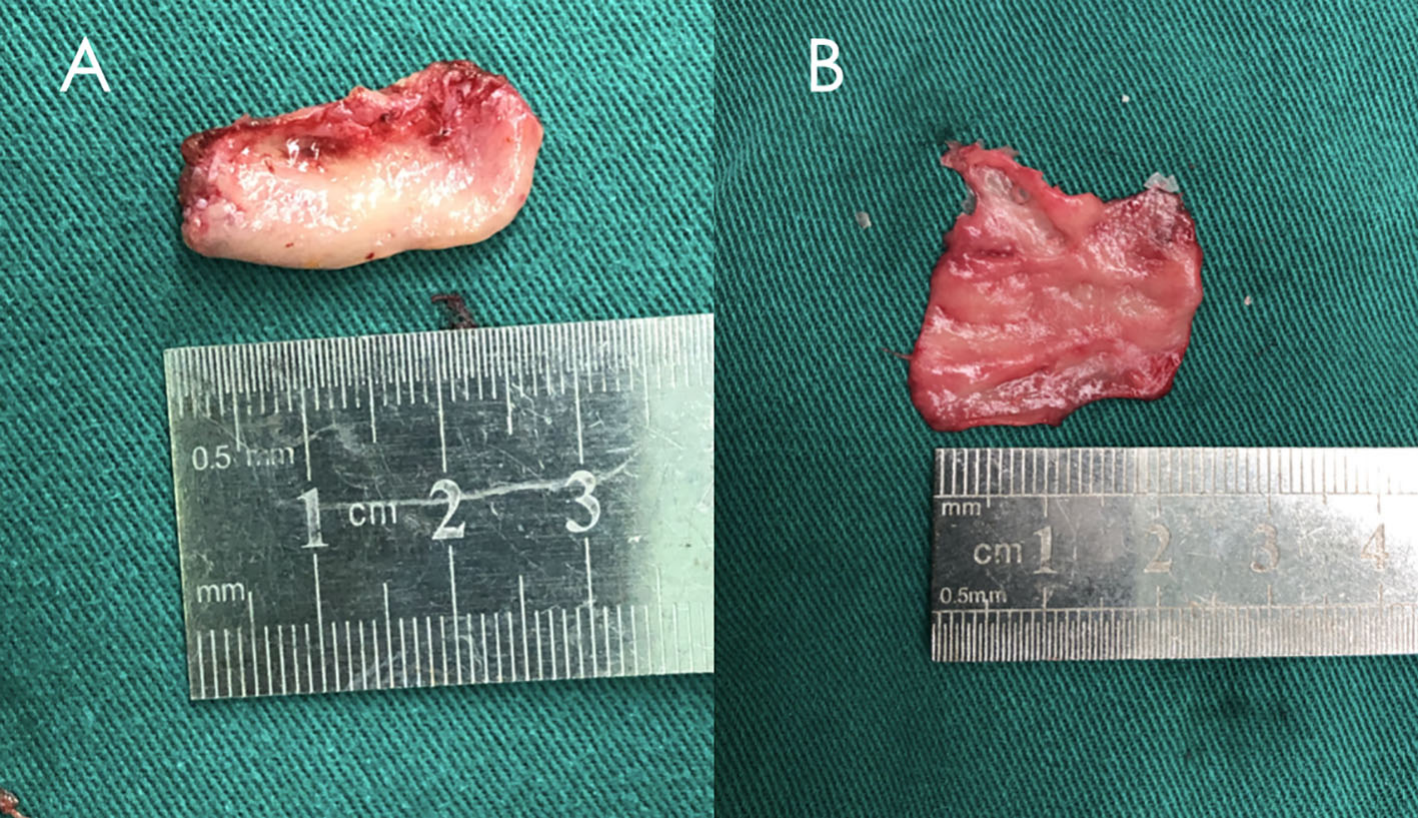
**A** middle turbinate was removed during surgery and the free middle turbinate mucosa flap was peeled off. **(A)** Middle turbinate. **(B)** Free middle turbinate mucosa flap.

**Figure 2:**
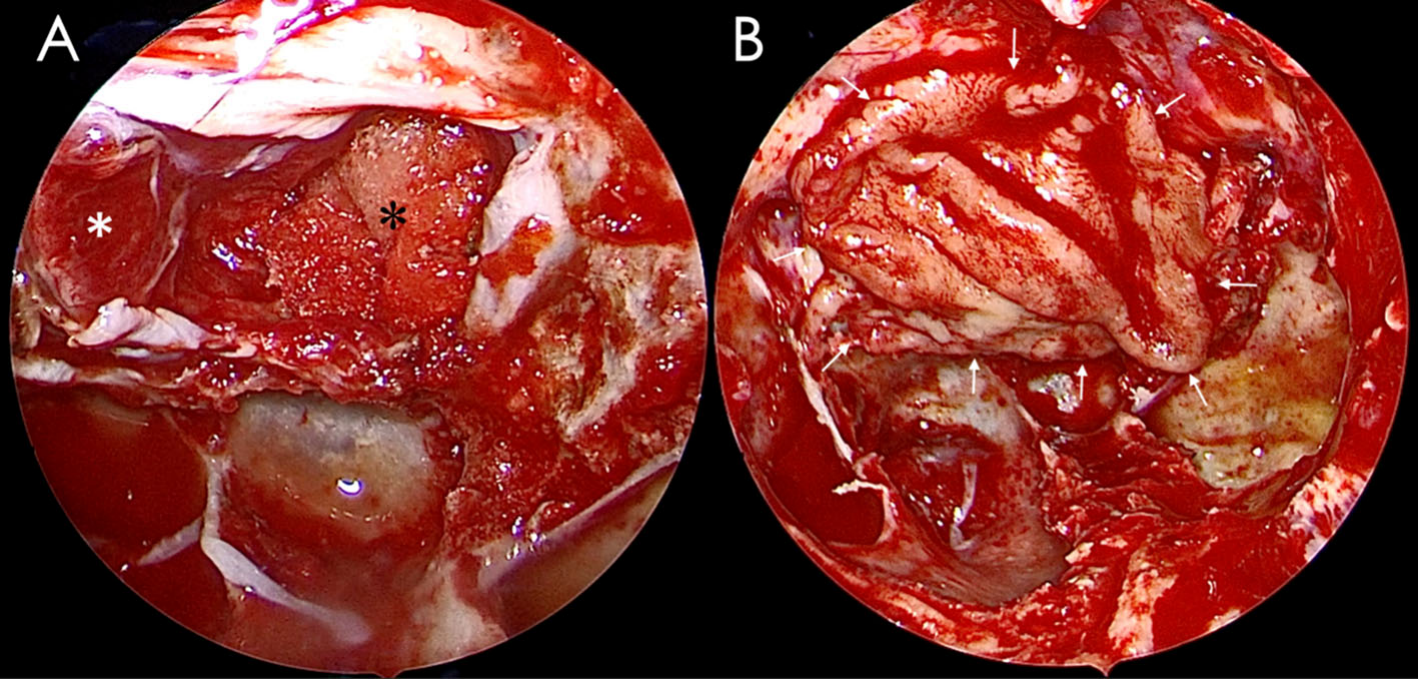
A free middle turbinate mucosa flap was used to reconstruct the sellar floor. **(A)** The pituitary tumor had been removed, and the defect of the sellar diaphragm was packed with gelfoam. **(B)** The sellar floor was repaired with the free middle turbinate mucosa flap. A white asterisk indicates the displaced pituitary gland, a black asterisk indicates the gelfoam, and a white arrow indicates the edge of the free middle turbinate mucosa flap.

Free nasal septum mucosa flap can be harvested in the surgery area. If we used the middle turbinate mucosa flap, it needs to be peeled off from the removed middle turbinate before use, as shown in **Figure 1**, paying attention to maintaining the integrity of the mucosa flap and avoiding damage as much as possible when we peel off the flap. No need to remove the bone of the middle turbinate. The free middle turbinate mucosa flap usually has a side length of approximately 2–3 cm, which is generally sufficient to repair the sellar floor defect after pituitary adenoma surgery, as shown in **Figure 1**. However, when encountering pituitary adenomas with significant enlargement of the sellar floor and patients with small middle turbinates, the free middle turbinate mucosal flap may not cover the dural gap of the sellar floor fully. Lay the free nasal mucosal flap flat on the dura defect of the skull base, completely covering it all around so that its edges come into contact with the surrounding bone, as shown in **Figure 2**.

Second, fascia lata was used in the following situations: there were apparent tears on the arachnoid membrane and sellar diaphragm, but the free nasal mucosa flap could not fully cover the dura defect of the skull base, or there was no available nasal mucosa flap that can be harvested in the nasal cavity due to the patient’s last surgery.

Third, Hadad–Bassagasteguy vascularized nasoseptal flap was used in the following situations: for invasive pituitary tumors, the suprasellar cistern or the third ventricular is exposed, resulting in high-flow cerebrospinal fluid leak; invasive pituitary tumors required open tuberculum sellae, planum sphenoidale; and the tumor invaded into the cavernous sinus, and the cavernous segment of the internal carotid artery was exposed after tumor removing. The method of vascularized nasoseptal flap harvesting had been described in the relevant literature (13).

The edge of the free nasal mucosa flap, fascia lata, or vascularized nasoseptal flap was covered with a small amount of hemostatic gauze and gelfoam and sprayed with biological protein glue.

If the free nasal mucosa flap or fascia lata was used, the nasal cavity should be packed with a sponge and removed 1–2 days after the surgery. If a vascularized nasoseptal flap was used, the sphenoid sinus and nasal cavity were packed with iodoform gauze and removed 7 days after the surgery. The lumbar drain is not used irregularly after surgery.

Within 1–2 months after surgery, patients were advised to avoid actions such as coughing, sneezing, defecating, bending, and lifting heavy objects that may instantly increase intracranial pressure.

## 3 Results

Complete removal of the tumor was considered to be gross total resection (GTR), more than 95% removal was considered to be near-total resection (NTR), 80%–95% removal was considered to be sub-total resection (STR), and <80% removal was considered to be partial resection (PR).

In the early period, of the 75 patients, GTR was achieved in 56 (74.7%) patients, NTR was achieved in 12 (16.0%) patients, STR was achieved in 6 (8.0%) patients, PR was achieved in 1 (1.3%) patient. The techniques used to reconstruct skull base in the late period group are shown in **Table 1**. Two (2.7%) patients had postoperative CSF leaks in this group. Intraoperative CSF leak existed in these two patients but without visible arachnoid defect. Inlayer fat graft and artificial dura matter were applied to repair the skull base for these two patients. One patient was cured by placing a lumbar drain, and one underwent operative repair twice and had meningitis, but the outcome was good.

**Table 1:** The skull base reconstruction techniques in the early stage.

Method	Intraoperative CSF leak did not exist	Intraoperative CSF leak exist	Number
**Artificial dura**	53	0	53 (70.7%)
**Fat graft and Artificial dura**	0	14	14 (18.7%)
**Fat graft, artificial dura, and fascia lata graft**	0	4	4 (5.3%)
**Fat graft, artificial dura, fascia graft, and pedicled nasoseptal flap**	0	4	4 (5.3%)
**Total**	53	22	75

In the late period, of the 125 patients, GTR was achieved in 105 (84.0%) patients, NTR was achieved in 3 (2.4%) patients, STR was achieved in 11 (8.8%) patients, and PR was achieved in 6 (4.8%) patients. The techniques used to reconstruct skull base in the late period group are shown in **Table 2**. No patient had postoperative CSF leak in this group.

**Table 2:** The skull base reconstruction techniques in the late stage.

Method	Intraoperative CSF leak did not exist	Intraoperative CSF leak exist	Number
**Free nasal septum mucosa flap**	18	3	21 (16.8%)
**Free middle turbinate mucosa flap**	46	38	84 (67.2%)
**Fascia lata graft**	0	2	2 (8.0%)
**pedicled nasoseptal flap**	7	11	18 (8.8%)
**Total**	71	54	125

## 4 Discussion

Skull base reconstruction is an essential step in EEA pituitary adenoma surgeries, especially in cases with intraoperative cerebrospinal fluid leak. Proper skull base reconstruction is crucial. Suppose the skull base cannot be properly repaired, it may require further surgery and even severe consequences such as secondary intracranial infection. There are significant differences in skull base repair methods among different authors. Regarding materials for skull base reconstruction, it includes various autologous materials, such as muscle patty, fat graft, fascia lata, free nasal mucosa flap, and pedicled nasal septal flap. Artificial materials include artificial dura mater and artificial bone. These reconstruction materials’ usage and applicability vary significantly among different authors.

The reconstruction materials can be divided into two categories based on placement and function. One type is materials placed intradural, such as muscle patty, fat graft, and artificial dura mater. Their primary function is to repair the skull base through subdural filling, which can prevent cerebrospinal fluid leak or at least reduce cerebrospinal fluid flow. Another type is placed epidural to achieve closure of skull base defects through adhesion and healing with the skull base bone and dura, such as fascia lata, free nasal mucosa flap, and pedicled nasal septal flap. Many surgeons use both types of materials for multi-layer repair simultaneously (14–16).

Free nasal mucosa flap, such as free middle turbinate mucosa flap and free nasal septum mucosa flap, can play a perfect role in the repair of the sellar floor, and the materials are easy to achieve (17, 18). Compared with the pedicled nasal septum flap, the free nasal mucosa flap has no severe side effects or nasal complications. Before the invention of the pedicled nasal septum flap, the free nasal mucosa flap played an important role in the repair of the sellar floor in EEA pituitary adenoma resection. The fascia lata is also a commonly used material for repairing the sellar floor during EEA pituitary adenoma resection, but the trauma of fascia lata harvesting is significant (19). Generally, it is used when there is no available free nasal mucosa or when the skull base defect is large and the free nasal mucosa flap cannot fully cover the defect of the skull base.

The Hadad–Bassagasteguy vascularized nasoseptal flap is a revolutionary technique for reconstructing the skull base. Applying a vascularized nasoseptal flap is very important if a high-flow cerebrospinal fluid leak exists (20). Harvesting the vascularized nasoseptal flap can lead to nasal complications. The majority of complications became manifest beyond the immediate postoperative period and were associated with the septal donor site, including septal perforation, prolonged crusting, and cartilage necrosis (21). It should not be applied to all patients with pituitary tumors. This study only harvested the pedicled vascularized nasoseptal flap in patients with a high-flow intraoperative CSF leak.

The intradural fat graft packing is a traditional method of skull base repair, which can be used alone or in combination with other materials mentioned above. Many surgeons use multiple layers of materials for skull base reconstruction, meantime filling fat graft intradurally (14, 16–19, 22). Intradural fat graft packing may completely seal the skull base, or at least reduce cerebrospinal fluid flow, and achieve skull base reconstruction by combining multiple layers repair (16, 23). We performed intradural fat graft packing in the early period group. Intradural fat graft packing was an effective method for repairing the skull base. However, there is still a certain probability of postoperative cerebrospinal fluid leak after intradural fat graft packing (24). Out of the 75 patients in our early period group, two suffered postoperative cerebrospinal fluid leak. Insufficient fat graft packing may not effectively seal the skull base. The volume of fat graft packed will gradually decrease after surgery (25). Insufficient filling and fat liquefaction absorption may be the reasons for postoperative cerebrospinal fluid leakage caused by only using fat graft to repair the skull base. Although many authors adopt intradural fat graft packing, there is no clear indication of the amount of fat graft used. Overpacking is also a potential risk; it may cause compression of the optic chiasm and optic nerve. Fat graft packing has its disadvantages. To harvest fat graft needs extra incision, and it may bring complications. Fat graft signal in the postoperative MRI can affect the evaluation of the extent of tumor resection and tumor recurrence.

Considering the side effects of fat graft packing, we changed the skull base reconstruction strategy to explore whether the fat graft packing is an indispensable material for the skull base repair in EEA pituitary adenoma resection in our late period group. Suppose there was no obvious intraoperative cerebrospinal fluid leak or only low-flow intraoperative cerebrospinal fluid leak. In that case, we used free autogenous materials such as the middle turbinate mucosa flap, nasal septum mucosa flap, and fascia lata. We used the pedicled nasal septum flap in case of a high-flow intraoperative cerebrospinal fluid leak or huge skull base defect. When using these autologous materials for the skull base repair, it was important to ensure that the skull base dura defect was completely covered, and the edges were in contact with the exposed skull base bone. We did not use fat graft or muscle patty for intradural packing. We used artificial dura mater intradural to reduce cerebrospinal fluid flow in all the cases. In our late period group, of the 125 patients, we completely abandoned intradural fat graft packing, and there was no postoperative cerebrospinal fluid leak. In our late period group, even if there was no obvious intraoperative cerebrospinal fluid leak, we still used the free nasal mucosa flap for routine skull base repair. Even if no apparent intraoperative cerebrospinal fluid leak was observed, the possibility of postoperative cerebrospinal fluid leak still existed (18, 23). The free nasal mucosa flap was harvested from the surgical area and had no severe side effects. At least one layer of reconstruction material was used in order to avoiding postoperative cerebrospinal fluid leak. The skull base reconstruction strategies that we adopted in the early and late periods had achieved good clinical results, indicating that intradural fat graft packing is not indispensable for the skull base reconstruction during EEA pituitary adenoma resection. Anyway, “less is more”; if intradural fat graft packing is not indispensable in preventing postoperative cerebrospinal fluid leak, we prefer to avoid this step.

## Data availability statement

The original contributions presented in the study are included in the article/supplementary material. Further inquiries can be directed to the corresponding authors.

## Ethics statement

The studies involving human participants were reviewed and approved by Institutional Ethics Committee of The First Affiliated Hospital of Nanjing Medical University. The patients/participants provided their written informed consent to participate in this study.

## Author contributions

CT and YY designed the study. CT and BW performed all the surgeries. XW and BW are responsible for data collection. XW and CT drafted the manuscript. YY, GC, and CT are responsible for revising and finalizing this paper. All authors contributed to the article and approved the submitted version.
